# Markov modeling in hepatitis B screening and linkage to care

**DOI:** 10.1186/s12976-017-0057-6

**Published:** 2017-05-18

**Authors:** Martin A. Sehr, Kartik D. Joshi, John M. Fontanesi, Robert J. Wong, Robert R. Bitmead, Robert G. Gish

**Affiliations:** 1 Department of Mechanical and Aerospace Engineering, University of California, San Diego, 9500 Gilman Drive, MS 0411, La Jolla, CA 92093-0411 USA; 2 Midwestern University, Arizona College of Osteopathic Medicine, 19555 North 59th Avenue, Glendale, AZ 85308 USA; 3 Department of Medicine, Division of General Internal Medicine, University of California, San Diego, 200 W. Arbor Drive #8415, San Diego, CA 92103 USA; 4 Division of Gastroenterology and Hepatology, Alameda Health System - Highland Hospital, 1411 East 31st Street, Highland Care Pavilion - 5th Floor Endoscopy Unit, Oakland, CA 94602 USA; 5 Department of Medicine, Division of Gastroenterology and Hepatology, Stanford University, Alway Building, Room M211, 300 Pasteur Drive, MC: 5187, Stanford, CA 94305-5187 USA; 6 National Viral Hepatitis Roundtable, 1612 K Street NW, Suite 1202, Washington, DC, 20006 USA; 7 Hepatitis B Foundation, 3805 Old Easton Road, Doylestown, PA USA

**Keywords:** Hepatitis B virus, Screening, Markov modeling, Point-of-care, Standard-of-care, Testing

## Abstract

**Background:**

With up to 240 million people chronically infected with hepatitis B worldwide, including an estimated 2 million in the United States, widespread screening is needed to link the infected to care and decrease the possible consequences of untreated infection, including liver cancer, cirrhosis and death. Screening is currently fraught with challenges in both the developed and developing world. New point-of-care tests may have advantages over standard-of-care tests in terms of cost-effectiveness and linkage to care. Stochastic modeling is applied here for relative utility assessment of point-of-care tests and standard-of-care tests for screening.

**Methods:**

We analyzed effects of point-of-care versus standard-of-care testing using Markov models for disease progression in individual patients. Simulations of large cohorts with distinctly quantified models permitted the assessment of particular screening schemes. The validity of the trends observed is supported by sensitivity analyses for the simulation parameters.

**Results:**

Increased utilization of point-of-care screening was shown to decrease hepatitis B-related mortalities and increase life expectancy at low projected expense.

**Conclusions:**

The results suggest that standard-of-care screening should be substituted by point-of-care tests resulting in improved linkage to care and decrease in long-term complications.

## Background

With up to 240 million people chronically infected with hepatitis B virus (HBV) worldwide [1], including an estimated 2 million people in the United States [2, 3], widespread testing to identify the infected is needed in order to link them to care and decrease the possible consequences of untreated HBV infection, which include approximately 500,000 to 1.2 million deaths yearly from liver cirrhosis and its complications, including primary liver cancer [1]. Limitations related to funding and access to commercially available tools for chronic HBV testing are particularly important in developing countries where the burden of chronic HBV is heaviest. Success of traditional standard-of-care (SOC) testing for HBV infection hinges on the existence of a systematic process of following up test results that return several days after testing, notifying patients of results, and arranging for follow-ups to discuss antiviral therapy, a system that requires resources that are limited in developing regions.

The development of rapid point-of-care (POC) tests for HBV has the potential to address many of these limiting factors and establish a more effective medical care model for chronic HBV. In a recent study of patients undergoing HBV screening, the performance characteristics of NanoSign® HBs POC chromatographic immunoassay was compared with standard commercial laboratory HBsAg testing (Quest Diagnostics EIA). The POC tests yielded a sensitivity of 73.7% and a specificity of 97.8% [4]. In a meta-analysis evaluating the accuracy of POC testing, Shivkumar et al. reported POC testing sensitivity of 93-98% and specificity of 93–99% [5]. Furthermore, the low cost ($0.50) and rapid turnaround (20 min from phlebotomy to test results) of POC tests give them the potential to significantly improve the widespread implementation of HBV screening, especially in resource-limited regions.

Modeling in HBV analysis and treatment is an active research topic and multiple approaches have been considered recently [6–8]. A variety of mathematical modeling strategies have been used to address in particular the cost-effectiveness of HBV screening, using predominantly combinations of decision trees and/or Markov chain models [9]. In this paper, we propose time-varying Markov chain models of detailed structure, reflecting disease propagation in individuals to quantify the effects of large-scale utilization of POC tests to succeed the SOC screening model.

## Methods

In comparing effectiveness of POC and SOC screening strategies for HBV, we made use of two Markov models with identical structure but different transition probabilities. Each of these models has six states capturing the medical progression of individual patients and is formed by aggregating states from a more detailed Markov model describing chronic HBV disease progression of individuals. The two aggregated models were used to simulate consequences of POC or SOC utilization in HBV screening strategies on large populations of individuals. Before they can be iterated numerically, the two Markov models rely on the specification of certain numerical values dealing with the rate of uptake of POC, the rate of infected patients seeking medical care, death rates, and so on. Some of these numbers can be determined (at least within a range) from the medical literature, which we did. Others are more hypothetical or might be the outcome of policy initiatives. The utility of the models is in their low computational cost and attendant capacity for iteration with many possible candidate values and the determination of the sensitivity of the observed behavior to the specific parameter values. Where available, the transition parameters in our models were selected from the literature. The remaining model parameters were estimated and their effects on the overall results analyzed in terms of sensitivity.

**Fig. 1:**
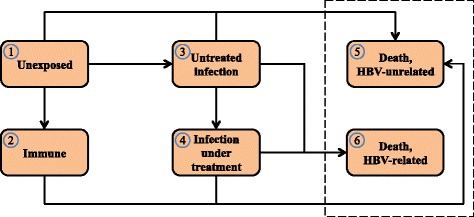
State transition diagram for aggregated Markov model. Connections illustrate feasible transitions per step. *Dashed box* encloses absorbing states

For the aggregated models, we considered the six patient states depicted in Fig. 1, where arrows symbolize state transitions admissible in a single time step. At year *t*, a patient is in state *i* with probability *π*_*i*,*t*_. Arranging these probabilities into a *state* row-vector, we have $$ {\varPi}_t=\left[\begin{array}{cccc}\hfill {\pi}_{1, t}\hfill & \hfill {\pi}_{2, t}\hfill & \hfill \dots \hfill & \hfill {\pi}_{6, t}\hfill \end{array}\right] $$. This vector is then propagated over time via *Π*_*t* + 1_ = *Π*_*t*_ *P*_*t*_, where *P*_*t*_ denotes the Markov state transition matrix at time *t* with element *p*_*ij*,*t*_ denoting the conditional probability of a patient in state *i* at year *t* transitioning to state *j* at year *t* + 1. Evidently, each row of the state transition matrix sums to one at all times. Special cases are *p*_*ij*,*t*_ = 0 for inadmissible transitions and *p*_*ij*,*t*_ = 1 for certain transitions. For instance, a transition from being immune to having an undetected HBV infection is inadmissible, whereas a deceased patient is going to remain so.

As illustrated by the connection between States 3 and 4 in Fig. 1, we assume that no patient starting treatment ever abandons this treatment. This assumption is justified in that it alters the transition probabilities *p*_36,*t*_ and *p*_46,*t*_ of dying from HBV with or without medical treatment by a relatively small degree, which is covered by the sensitivity analysis described below. Model States 3 and 4 in this model are aggregated states from more detailed Markov models described below. This common model structure for both SOC and POC screening policies takes *P*_*t*_ of form

$$ {P}_t=\left[\begin{array}{cccccc}\hfill {p}_{11, t}\hfill & \hfill {p}_{12, t}\hfill & \hfill {p}_{13, t}\hfill & \hfill 0\hfill & \hfill {p}_{15, t}\hfill & \hfill 0\hfill \\ {}\hfill 0\hfill & \hfill {p}_{22, t}\hfill & \hfill 0\hfill & \hfill 0\hfill & \hfill {p}_{25, t}\hfill & \hfill 0\hfill \\ {}\hfill 0\hfill & \hfill 0\hfill & \hfill {p}_{33, t}\hfill & \hfill {p}_{34, t}\hfill & \hfill {p}_{35, t}\hfill & \hfill {p}_{36, t}\hfill \\ {}\hfill 0\hfill & \hfill 0\hfill & \hfill 0\hfill & \hfill {p}_{44, t}\hfill & \hfill {p}_{45, t}\hfill & \hfill {p}_{46, t}\hfill \\ {}\hfill 0\hfill & \hfill 0\hfill & \hfill 0\hfill & \hfill 0\hfill & \hfill 1\hfill & \hfill 0\hfill \\ {}\hfill 0\hfill & \hfill 0\hfill & \hfill 0\hfill & \hfill 0\hfill & \hfill 0\hfill & \hfill 1\hfill \end{array}\right]=\left[\begin{array}{cccccc}\hfill {p}_{11, t}\hfill & \hfill {\boldsymbol{p}}_{12}\hfill & \hfill {p}_{13}\hfill & \hfill 0\hfill & \hfill {p}_{5, t}\hfill & \hfill 0\hfill \\ {}\hfill 0\hfill & \hfill {p}_{22, t}\hfill & \hfill 0\hfill & \hfill 0\hfill & \hfill {p}_{5, t}\hfill & \hfill 0\hfill \\ {}\hfill 0\hfill & \hfill 0\hfill & \hfill {p}_{33, t}\hfill & \hfill {\boldsymbol{p}}_{34}\hfill & \hfill {p}_{5, t}\hfill & \hfill {p}_{36}\hfill \\ {}\hfill 0\hfill & \hfill 0\hfill & \hfill 0\hfill & \hfill {p}_{44, t}\hfill & \hfill {p}_{5, t}\hfill & \hfill {p}_{46}\hfill \\ {}\hfill 0\hfill & \hfill 0\hfill & \hfill 0\hfill & \hfill 0\hfill & \hfill 1\hfill & \hfill 0\hfill \\ {}\hfill 0\hfill & \hfill 0\hfill & \hfill 0\hfill & \hfill 0\hfill & \hfill 0\hfill & \hfill 1\hfill \end{array}\right], $$

where *p*_12,*t*_ = ***p***_12_ and *p*_34,*t*_ = ***p***_34_ are constant transition probabilities chosen depending on the screening strategy at hand. The constant transition probability *p*_13_ is presumed independent of the screening policies while *p*_36_ and *p*_46_ are results of the aggregation procedure outlined below. Time-variation of the state transition matrix *P*_*t*_ is caused solely by the varying propensity for death, *p*_5,*t*_ as age advances, which is modeled through linear inter- and extrapolation of annual mortality rates for individuals in the USA [10]. However, even though all time variations are induced by variations in *p*_5,*t*_, notice that the changing mortality rates affect the first four transition probabilities on the diagonal of the state transition matrix, being the probabilities to remain in the respective non-absorbing states. For illustration, whenever *p*_5,*t*_ increases at some time, *p*_22,*t*_ has to decrease by just as much to ensure that the sum of all transition probabilities from State 2 is one at all times *t*. That is, we require *p*_22,*t*_ = 1 − *p*_5,*t*_ for all times *t*. These time-adjustments have to be made for all rows of the state transition matrix corresponding to non-absorbing states (i.e., the first four rows of *P*_*t*_).

Special cases comprise the third and fourth rows of *P*_*t*_, which capture transitions emerging from the two aggregated states corresponding to HBV disease progression in untreated and treated forms, respectively. We next discuss aggregation of a more complex model capturing the natural history of chronic HBV to estimate the transition probabilities corresponding to State 3 and 4 of the aggregated model in Fig. 1.

**Fig. 2:**
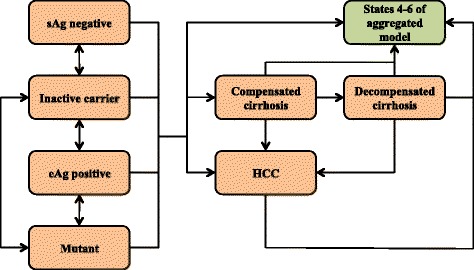
Disease model for untreated chronic HBV. State transition diagram of disease model for untreated chronic HBV, expanding State 3 in aggregated Markov model. Connections illustrate feasible transitions per step. Absorption to States 4–6 in aggregated Markov model via *green* box

To form the aggregation resulting in State 3, *untreated infection,* we captured the disease progression of HBV without treatment using a different Markov model with its own states and admissible transitions as depicted in Fig. 2. Transition probabilities in this model are based on a literature review and subsequent weighting of the annual probabilities reported in the references using the GRADE criteria [11] for assessing the quality of each study. The resulting transition probabilities are summarized in Table 1, where HCC connotes hepatocellular carcinoma (liver cancer). We refer to the auxiliary model corresponding to Fig. 2 equipped with annual transition probabilities summarized in Table 1 as the *disease model*, while we refer to the more compact six-state model described above as the *aggregated model*.

**Table 1:** Annual transition probabilities for untreated chronic HBV infection

From	To	Probability	References
eAg+	Inactive carrier	7.67%	[13–18]
	Mutant	1.90%	[14–16]
	Compensated cirrhosis	2.96%	[14–18]
	HCC	0.92%	[13–17]
Inactive carrier	Mutant	6.16%	[15, 17, 18]
	sAg-	0.55%	[15, 17]
	eAg+	2.54%	[17]
	Compensated cirrhosis	0.93%	[13, 15, 17]
	HCC	0.35%	[15, 17]
	HBV-related death	0.04%	[19]
Mutant	Inactive carrier	1.60%	[14–16]
	eAg+	20.05%	[20]
	Compensated cirrhosis	4.93%	[13–18]
	HCC	0.60%	[15, 17]
	HBV-related death	0.60%	[15]
sAg-	Inactive carrier	13.40%	[21]
	Compensated cirrhosis	0.18%	[22, 23]
	HCC	0.15%	[23, 24]
Compensated cirrhosis	Decompensated cirrhosis	5.08%	[13–18, 25–27]
	HCC	3.02%	[13–18, 25–27]
	HBV-related death	4.29%	[13–16, 18, 26, 27]
Decompensated cirrhosis	HCC	5.94%	[13, 15, 17, 18, 27]
	HBV-related death	21.46%	[13–18, 25–27]
HCC	HBV-related death	40.11%	[13–18, 25–27]

Absorption in the disease model means transition to the union of the States 4 (Infection under treatment), 5 (death, HBV-unrelated) and 6 (death, HBV-related) of the aggregated model, which can be reached from any state in the disease model.

1. Initiation of treatment occurs via *p*_34,*t*_ = ***p***_34_ defined for the aggregated model above.
2. Death unrelated to HBV in the disease model is assumed time-invariant with annual probability of 0.1%, corresponding to an individual of age 25–34 years [10].
3. HBV-related death follows the probabilities listed in Table 1.

The total absorption probability at any state in the disease model is then the sum of the three aforementioned component probabilities. Having the purpose of aggregation in mind, we do not need to distinguish the absorbing states in the disease model any further. The technical tool used to aggregate the disease model into State 3 of the aggregated model is the *fundamental matrix N* = (*I* − *Q*)^− 1^ of the disease model, where *Q* is the matrix obtained by extracting all rows and columns of the state transition matrix corresponding to transient states. The fundamental matrix *N* allows a number of useful deductions about the Markov chain, such as expected numbers of occupancies in transient states until absorption and corresponding variances.

A particularly useful property of the fundamental matrix is that element *n*_*ij*_ equals the expected number of years spent at state *j* of the disease model when starting in state *i* [12]. That is, row *i* of *N* accumulates the expected numbers of years in each disease state given the process is initiated in state *i*. Given a sufficiently large number of patients, the normalized version of this row-vector can be interpreted as the average fraction of time spent at each state until absorption, where normalization refers to scaling the vector such that its components have sum one. We can now estimate the probability of death caused by untreated HBV by forming this normalized vector from the first row of the fundamental matrix and using its components to obtain a weighted sum of the probabilities for HBV-related death in Table 1. This weighted sum is used for *p*_36_, concluding the aggregation of the disease model into State 3 of the aggregated model. Assuming probabilities ***p***_34_ = 15% of initiating medical treatment and 0.1% for HBV-unrelated death in the disease model, this procedure yields the estimate *p*_36_ = 1.35%. Notice that this probability depends implicitly on the screening policy employed via variation of ***p***_34_.

State 4, *Infection under treatment* of the aggregated model can be viewed as an aggregation of the same states used to form the disease model (Fig. 2), although with annual transition probabilities differing from those summarized in Table 1 to reflect effects of treatment. Moreover, the probability of absorption from this disease model under treatment would be decreased by the amount of ***p***_34_. The effect of these transition probabilities for the disease model under treatment is a value for the HBV-related mortality rate under treatment in the aggregated model, *p*_46_. To obtain this transition probability, we correct the probability for absorption in the disease model used above by ***p***_34_, but keep using the values in Table 1. To adjust for the favorable effects of medical intervention, we introduce a scaling parameter *α* ∈ (0, 1) and estimate *p*_46_ = *αp*_46_^*^, where *p*_46_^*^ denotes the probability obtained after aggregation with the values in Table 1. For instance, scaling factor *α* = 0.25 and fixed annual probability 0.1% for HBV-unrelated death result in the estimate *p*_46_ = 0.54%. This scaling approach is chosen as we focus on effects of screening policies rather than treatment options. Summarizing the modeling and aggregation procedure, the state transition matrix for the aggregated models takes on the structure

$$ {P}_t=\left[\begin{array}{cccccc}\hfill 1-\left({\boldsymbol{p}}_{12}+{p}_{13}+{p}_{5, t}\right)\hfill & \hfill {\boldsymbol{p}}_{12}\hfill & \hfill {p}_{13}\hfill & \hfill 0\hfill & \hfill {p}_{5, t}\hfill & \hfill 0\hfill \\ {}\hfill 0\hfill & \hfill 1-{p}_{5, t}\hfill & \hfill 0\hfill & \hfill 0\hfill & \hfill {p}_{5, t}\hfill & \hfill 0\hfill \\ {}\hfill 0\hfill & \hfill 0\hfill & \hfill 1-\left({\boldsymbol{p}}_{34}+{p}_{5, t}+{p}_{36}\right)\hfill & \hfill {\boldsymbol{p}}_{34}\hfill & \hfill {p}_{5, t}\hfill & \hfill {p}_{36}\hfill \\ {}\hfill 0\hfill & \hfill 0\hfill & \hfill 0\hfill & \hfill 1-\left({p}_{5, t}+\alpha {p}_{46}^{*}\right)\hfill & \hfill {p}_{5, t}\hfill & \hfill \alpha {p}_{46}^{*}\hfill \\ {}\hfill 0\hfill & \hfill 0\hfill & \hfill 0\hfill & \hfill 0\hfill & \hfill 1\hfill & \hfill 0\hfill \\ {}\hfill 0\hfill & \hfill 0\hfill & \hfill 0\hfill & \hfill 0\hfill & \hfill 0\hfill & \hfill 1\hfill \end{array}\right], $$

with HBV-unrelated mortality rates *p*_5,*t*_ from the literature [10] and the transition probabilities in the third and fourth rows depending on the aggregation procedure outlined above. In the following, we use this transition matrix structure for simulation and corresponding sensitivity analyses based on a number of constants in the state transition matrix, namely the transition probabilities **p**_12_, p_13_, **p**_34_ and the scaling parameter α. As mentioned above, the effects of SOC and POC screening strategies are compared using altered transition probabilities ***p***_12_ and ***p***_34_ in the aggregated model, which in terms also affects the state aggregation yielding *p*_36_ as well as the respective transition probabilities on the diagonal of *P*_*t*_. Higher utilization of POC screening with subsequent immunization in uninfected cases and initiation of medical treatment in infected cases, respectively, is anticipated to increase both ***p***_12_ and ***p***_34_, albeit to different degrees. To model these changes, we take ***p***_12_ → *p*_12_^*SOC*^ and ***p***_34_ → *p*_34_^*SOC*^ in the SOC case. In the POC case, we take ***p***_12_ → *p*_12_^*POC*^ = *βp*_12_^*SOC*^ and ***p***_34_ → *p*_34_^*POC*^ = *γp*_34_^*SOC*^, employing additional scaling parameters *β* and *γ*, each greater than one.

The approach taken to analyze POC/SOC utilization effectiveness using the quantified aggregated model is to model a population of a large number of individuals starting with an initial probability distribution over the six states and then to propagate the Markov chain until the collective probability of the death states is nearly one. We presume the population comprises 100,000 initially uninoculated and uninfected 10-year-olds and the evolution of the Markov chain over time yields the anticipated proportions of the aging population in each state. The assessment of various performance measures such as mortality rates, years under treatment or life expectancy under SOC and POC screening policies is then tracked via evolution of the probability vector *Π*_*t*_, which now admits the interpretation as the proportions of the population occupying each disease state, since the population is presumed large.

As mentioned above, we use an annual probability of 0.1% for HBV-unrelated death in the disease model. The remaining transition probabilities in the disease model are according to Table 1. The time-varying probabilities for HBV-unrelated death in the aggregated model, *p*_5,*t*_, are obtained via inter- and extrapolation of mortality data for individuals in the U.S. [10]. The remaining simulation parameters to be chosen are the scaling constants *α*, *β* and *γ* as well as the transition probabilities p_13_, *p*_12_^*SOC*^ and *p*_34_^*SOC*^. In the following, we use the nominal parameter values *p*_12_^*SOC*^ = 0.2%, *p*_34_^*SOC*^ = 15%, *p*_13_ = 0.15%, *α* = 0.25, *β* = 5 and *γ* = 2 unless otherwise specified. Sensitivity analyses around the nominal parameter values specified above are displayed in Table 2, one for the inoculation-related parameters *p*_12_^*SOC*^ and *β*, one for the treatment-related parameters *p*_34_^*SOC*^ and *γ* and one for the screening-unrelated parameters *p*_13_ and *α*. Each sensitivity analysis uses nominal values for the remaining parameters.

**Table 2:** Sensitivity data for simulation parameters

Sensitivity Parameters	HBV-caused deaths			Life expectancy, years		
	SOC	POC	Drop	SOC	POC	Rise
*α* = 0.20						
*p*_13_ = 0.10%	11182	7649	31.60%	77.87	77.97	0.13%
*p*_13_ = 0.15%	16556	11347	31.46%	77.72	77.87	0.19%
*p*_13_ = 0.20%	21791	14963	31.33%	77.57	77.77	0.26%
*α* = 0.25						
*p*_13_ = 0.10%	12981	9356	27.93%	77.83	77.93	0.13%
*p*_13_ = 0.15%	19224	13879	27.80%	77.66	77.80	0.18%
*p*_13_ = 0.20%	25309	18303	27.68%	77.49	77.68	0.25%
*α* = 0.30						
*p*_13_ = 0.10%	14682	10962	25.34%	77.79	77.88	0.12%
*p*_13_ = 0.15%	21746	16262	25.22%	77.60	77.74	0.18%
*p*_13_ = 0.20%	28633	21447	25.10%	77.41	77.60	0.25%
*β* = 4						
*p*_12_^*SOC*^ = 0.15%	19472	15193	21.98%	77.65	77.78	0.17%
*p*_12_^*SOC*^ = 0.20%	19224	14512	24.51%	77.66	77.79	0.17%
*p*_12_^*SOC*^ = 0.25%	18981	13879	26.88%	77.66	77.80	0.18%
*β* = 5						
*p*_12_^*SOC*^ = 0.15%	19472	14678	24.62%	77.65	77.79	0.18%
*p*_12_^*SOC*^ = 0.20%	19224	13879	27.80%	77.66	77.80	0.18%
*p*_12_^*SOC*^ = 0.25%	18981	13147	30.74%	77.66	77.82	0.21%
*β* = 6						
*p*_12_^*SOC*^ = 0.15%	19472	14190	27.13%	77.65	77.80	0.19%
*p*_12_^*SOC*^ = 0.20%	19224	13288	30.88%	77.66	77.82	0.21%
*p*_12_^*SOC*^ = 0.25%	18981	12475	34.28%	77.66	77.83	0.22%
*γ* = 1.5						
*p*_34_^*SOC*^ = 10%	21843	15838	27.49%	77.57	77.73	0.21%
*p*_34_^*SOC*^ = 15%	19224	14484	24.66%	77.66	77.78	0.15%
*p*_34_^*SOC*^ = 20%	17910	13879	22.51%	77.70	77.80	0.13%
*γ* = 2.0						
*p*_34_^*SOC*^ = 10%	21843	14811	32.19%	77.57	77.77	0.26%
*p*_34_^*SOC*^ = 15%	19224	13879	27.80%	77.66	77.80	0.18%
*p*_34_^*SOC*^ = 20%	17910	13494	24.66%	77.70	77.82	0.15%
*γ* = 2.5						
*p*_34_^*SOC*^ = 10%	21843	14233	34.84%	77.57	77.79	0.28%
*p*_34_^*SOC*^ = 15%	19224	13564	29.44%	77.66	77.82	0.21%
*p*_34_^*SOC*^ = 20%	17910	13305	25.71%	77.70	77.83	0.17%

Population size 100,000; initial age 10 years; unless otherwise noted per row: ***p***_12_^***SOC***^ = 0.2%, ***p***_34_^***SOC***^ = 15%, ***p***_13_ = 0.15%, ***α*** = 0.25, ***β*** = 5 and ***γ*** = 2

## Results

**Fig. 3:**
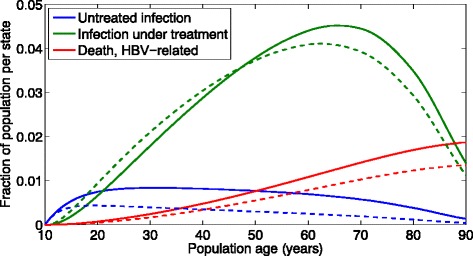
Expected occupancies of States 3, 4 and 6 in the aggregated Markov model. *Solid lines* = standard of care (SOC); *dashed lines* = point of care (POC); initial cohort age of 10 years; parameter values: chance of immunization, SOC, *p*_12_^*SOC*^ = 0.2%; chance of treatment initiation, SOC, *p*_34_^*SOC*^ = 15%; chance of infection, *p*_13_ = 0.15%; treatment effectiveness factor, *α* = 0.25; immunization factor, *β* = 5; treatment initiation factor, *γ* = 2. *Solid lines* for SOC, *dashed lines* for POC; initial cohort age of 10 years

Simulation results based on our cohort of 100,000 initially uninfected and uninoculated 10-year-olds and the nominal simulation parameters specified above are displayed in Fig. 3. As anticipated, utilization of POC screenings reduces the numbers of untreated infections and HBV-related mortalities at all times. This holds for all setups of the simulation parameters, as long as the scaling parameters are confined to their respective boundaries, that is 0 < *α* < 1, *β* > 1 and *γ* > 1. The number of infections under treatment using POC screening is initially higher but lower in average than with SOC screening. This trend is a result of both the increased inoculation rate *p*_12_^*POC*^ > *p*_12_^*SOC*^ and the increased rate for initiation of treatment *p*_34_^*POC*^ > *p*_34_^*SOC*^. Initially, about the same number of people gets infected under each screening strategy, while more of those infected individuals are linked to medical treatment in the POC case. As the population ages, a larger fraction of the cohort has been inoculated in the POC case, which results in a decreased number of new infections. This in turns leads to a smaller number of patients with untreated infections that can potentially be linked to care, resulting in lower average numbers of patients receiving medical treatment under the POC screening setup. However, the ratio of people linked to care over those infected without treatment is significantly higher in the POC case. The results from our modeling serve to quantify and bound these results, which are a logical consequence of the model’s structure.

The data displayed in Fig. 3 are based on the particular set of nominal parameter values specified above, with the resultant findings extended to different parameter combinations. Sensitivity analyses around the nominal parameter values specified above are displayed in Table 2 for the following varied parameter combinations: the inoculation-related parameters *p*_12_^*SOC*^ and *β*; the treatment-related parameters *p*_34_^*SOC*^ and *γ*; and the screening-unrelated parameters *p*_13_ and *α*. For presentation purposes, each sensitivity analysis presumes nominal values for the remaining simulation parameters. However, the trends summarized in the following paragraphs extend to combined sensitivity analysis. The indicators listed to evaluate the simulations are the total numbers of HBV-related mortalities and life expectancies under POC and SOC screening policies as well as relative improvements gained by implementing POC screenings. For instance, the nominal parameter values result in improvements of 27.8% in HBV-related mortality numbers 0.18% in life expectancy, respectively. Sensitivity is interpreted as variation in these two relative measures for the benefit in POC screening utilization. The reason for the seemingly low changes in life expectancy is that only a fraction of the population ever gets infected with HBV, while the change in life expectancy for infected individuals is larger.

In the first sensitivity analysis, only the treatment effectiveness factor *α* and the infection rate *p*_13_ vary from their nominal values. These are the simulation parameters presumed independent of the screening policies employed. As we can see, each of the tested combinations of these two parameters yields improvements of at least 25.1% in total HBV-related death numbers and 0.12% in life expectancy in the POC screening case. In general, we observe trends for larger improvements in HBV-related mortality numbers towards higher treatment effectiveness (i.e., lower value for *α*). The infection rate *p*_13_ has only minor influence on the improvement in HBV-related death totals, while increasing the gains in life expectancy at higher infection rates. The comparatively small influence of *p*_13_ on the relative improvements in mortality numbers is not surprising as *p*_13_ only changes the proportions of the population ever to become infected, but not the change of course for patients after being infected. The treatment effectiveness factor *α*, however, is strongly linked to potential gains in POC screening by the improved linkage to care and thus affects relative improvements in mortality numbers to a greater extent. The reason for the strong sensitivity of the gains in life expectancy to the infection rate is that if a larger fraction of the population becomes infected, the relative weight of the improvements for this fraction on the entire population grows.

The second sensitivity study focuses on variations of the inoculation-related simulation parameters *p*_12_^*SOC*^ and *β*. Using the parameter values in Table 2, we gain improvements of at least 21.98% in mortality numbers and at least 0.17% in life expectancies when implementing POC screenings. As expected, both scaling factor *β* and base inoculation rate *p*_12_^*SOC*^ have significant influence on the two measures of improvement obtainable using POC tests. However, even for low scaling factors *β* and high base inoculation rates, notable benefits of POC test utilization are apparent. In the third sensitivity study, the treatment-related simulation parameters *p*_34_^*SOC*^ and *γ* are varied. Improvements are at least 22.51% in HBV-related mortality numbers and 0.13% in life expectancy, while both parameters appear to have similar influence on the two measures.

## Discussion and conclusions

Chronic HBV is a worldwide problem, with millions of new people infected each year and a large population of chronically infected patients facing health care consequences both short- and long-term. However, many chronic HBV patients remain asymptomatic and millions worldwide are unaware of their infections. The importance of early detection via HBV screening of high-risk individuals hinges on the ability to implement effective antiviral therapy to prevent progression of liver disease leading to complications such as cirrhosis and hepatocellular carcinoma. While commercially available serologic immunoassays are widely used for HBV screening, the availability and access to these testing tools for resource-limited regions or marginalized populations such as the homeless and immigrants are suboptimal. Furthermore, the effort associated with following up on SOC test results, patient call-back and counseling can be considerable and create further hurdles for implementing effective screening programs. Recent development of POC tests for HBV holds promise, and previous studies have reported satisfactory sensitivity and specificity of POC testing when compared with SOC testing. However, few studies have used a modeling approach that not only takes into account the performance characteristics of POC testing, but also the natural history of untreated HBV infection to evaluate accurately the added benefit of POC testing over SOC testing. Given the significantly lower cost and more rapid turnaround time associated with POC testing for HBV, the replacement of SOC testing by POC testing has the potential to improve HBV screening programs by promoting greater access and improving linkage to care.

Using Markov modeling based on a comprehensive literature review, our current study demonstrates that POC testing is associated with significantly lower HBV-related mortality and greater life expectancy when compared with SOC testing. In conclusion, the simulation results under various parameter selections indicate that a significant improvement is obtainable via replacement of SOC screening by new POC tests. The clinical impact of POC testing may be even greater in resource-limited regions and among marginalized populations where health care access and follow-up after testing are obstacles to the effective implementation of HBV screening programs. In a future study, additional measures such as morbidity and expected cost of treatment will be analyzed based on additional data regarding cost and effectiveness of medical treatment as well as costs of POC and SOC screening implementation.

## Abbreviations

HBV: Hepatitis B virus
POC: Point-of-care
SOC: Standard-of-care
